# mRNA localization and local translation in neurons

**DOI:** 10.3934/Neuroscience.2020016

**Published:** 2020-08-10

**Authors:** Mohammad Mofatteh

**Affiliations:** 1 Lincoln College, University of Oxford, Turl Street, Oxford, OX1 3DR, United Kingdom; 2 Merton College, University of Oxford, Merton Street, Oxford, OX1 4DJ, United Kingdom; 3 Sir William Dunn School of Pathology, University of Oxford, South Parks Road, Oxford, OX1 3RE, United Kingdom

**Keywords:** mRNA localization, local translation, neuron, nervous system, neuronal development

## Abstract

The spatial and temporal regulation of gene expression in neurons is an important step in creating functional and structural neuronal networks. The complexity of neurons require differential expression of various proteins in different compartments. Highly polarised cells, such as neurons, respond rapidly to different external stimuli by changing their local protein abundance and composition. Neurons can have extensions up to a meter away from their cell body in humans, so it is easy to envisage why they need to manage the synthesis of new proteins locally and on-demand. Recent research has demonstrated that neurons can control the expression of different proteins by localising translationally silent mRNAs, followed by subsequent translation. Neurons use mRNA localization and local translation to achieve different purposes during their life cycle. While developing neurons rely on mRNA localization for axon guidance and synaptogenesis, mature neurons can use mRNA localization for maintenance of essential physiological processes. mRNA localization also plays a role in response to neuron injury to regenerate and restore neuronal connections. Recent microscopic imaging techniques such as live imaging of fluorescently tagged molecules combined with genetic and biochemical studies in neurons have illustrated evolutionarily conserved mechanisms for targeting mRNAs into their correct compartments. This review provides an overview of mRNA localization and local translation in vertebrate and invertebrate neurons and discusses the mechanism by which mRNAs are trafficked into axons. Furthermore, the role of mRNA localization in synaptic activation, as well as axonal injury is explored.

## 1. Introduction

**Figure 1.**
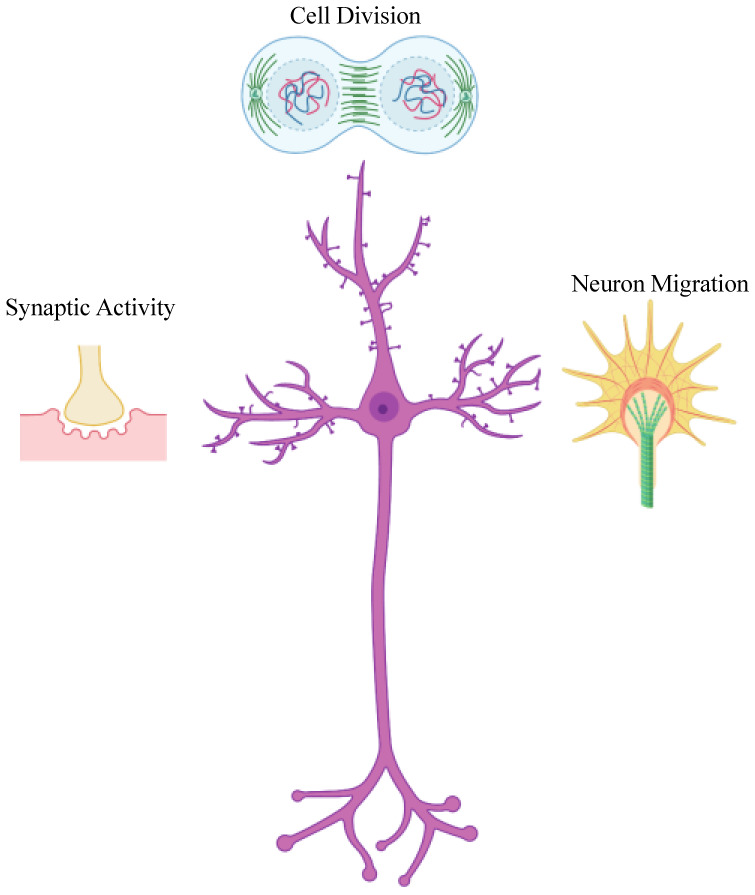
mRNA localization plays significant role in multiple aspects of neuronal development and maintenance. mRNA localization coupled to local translation is important in various events, such as neuroblast division, axon guidance and synaptogenesis, and sustained neuronal activities in developing and mature neurons.

Since the discovery of polyribosomes at the bases of dendritic spines by Steward and Levy in 1982, many lines of evidence have demonstrated that mRNA localization and *de novo* synthesis of proteins in the nervous system are critical steps for various nervous system processes such as development of neurons, synaptic plasticity and long-term memory formation [1],[2]. mRNA localization plays a significant role in central and peripheral nervous systems such as neuronal cell division and neuroblast maintenance, neuron migration, and synaptic activity (Figure 1) [3].

Many aspects of neuron behaviour and signalling in the central nervous system (CNS) require spatial control over the distribution of proteins. The positioning of proteins to different regions of the neuron can determine their function. The common depiction in the central dogma of molecular biology that mRNAs are simply transcript molecules for transferring nuclear information in the DNA to the protein synthesis machinery in the cytoplasm has changed. Local control over the expression of genes can be achieved by trafficking mRNA molecules to different subcellular compartments, where they are translated to their protein products. Thus, mRNAs can have active roles in regulating protein expression through their subcellular localization and local translation. However, mRNA trafficking and protein trafficking are not mutually exclusive mechanisms for subcellular localization of cargoes in the cytoplasm. mRNA localization can work in parallel to the protein sorting machinery, ensuring efficient targeting of proteins to their site of action.

The first evidence for asymmetric mRNA localization in the cytoplasm was found in an ascidian, *Styela plicata* about four decades ago. *In situ* hybridization using a DNA probe showed that *actin* mRNA was asymmetrically distributed in different regions of the cytoplasm of the ascidian embryo, with the highest concentration in the myoplasm and the lowest concentration in the endoplasm [4]. Since then, it has been shown that various systems such as *Saccharomyces cerevisiae*, migrating fibroblasts, *Caenorhabditis elegans*, *Drosophila melanogaster*, *Xenopus laevis*, epithelial cells, neuroblasts, and nerve cells can localize mRNAs [5]–[12]. Observations of asymmetrical distribution of mRNAs in different systems and across different species indicate that subcellular localization of mRNAs is a prevalent and evolutionarily conserved mechanism to distribute proteins to different regions of the cell.

## 2. The importance of mRNA localization and local translation in the nervous system

Data from deep sequencing and high-resolution imaging of rat hippocampal neurons led to the conclusion that approximately half of neuronally expressed mRNAs are enriched in dendrites compared to the soma [13]. A significant portion of these mRNAs encode for synaptic proteins such as receptors, scaffolding factors, and proteins involved in signalling pathways.

**Figure 2.**
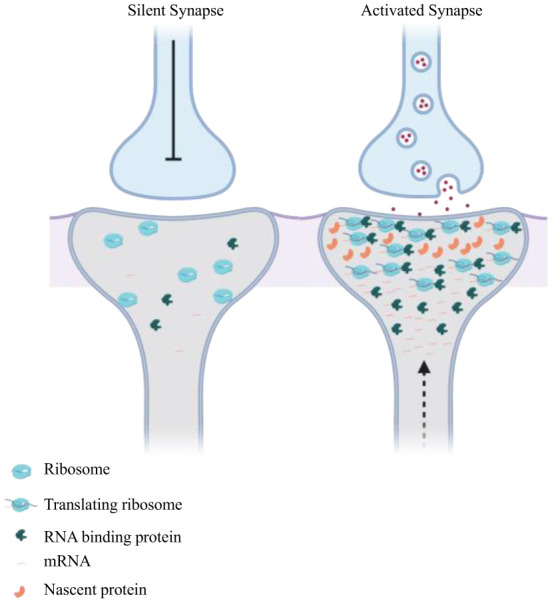
Neuronal stimulation drives mRNA localization in dendrites. Synaptic activity can induce reversible localization and local translation of certain mRNAs into active dendritic spines. The base of dendritic spine can act as a hub to accumulate localizing mRNAs which translocate into dendritic spine upon neuronal firing.

Localization of mRNA and subsequent translation can be regulated by neuronal activity (Figure 2). *Calcium/calmodulin-dependent kinase-2α* (*CamKIIα*) co-localizes *in vivo* with the RNA localization factor Staufen in dendrites of hippocampal neurons upon depolarisation with potassium chloride (KCl), and the RNA has a stronger enrichment in dendrites upon neuronal stimulation [14]–[16]. *β-actin* mRNA and its associated proteins can localize in dendritic spines upon neuronal stimulation. Live imaging of a GFP-tagged version of the *β-actin* mRNA binding protein ZBP1 showed that proteins redistributed from the cell bodies to the base and spines of dendrites of hippocampal neurons upon depolarisation with KCl [17]. The activity-dependent relocalization of *ZBP1* was abolished after treatment of neurons with antagonists of NMDA receptors [17]. Also, microtubule-dependent localization of mRNAs encoding the neurotrophin BDNF and its receptor TrkB to dendrites of hippocampal neurons increased two-fold when the neurons were stimulated [18]. Depolarizing cultures of neurons and acute brain slices with KCl elicited a significant increase in the density of *β-actin* mRNA granules in dendrites [19]. However, it is still unknown whether the activity-dependent increase in mRNA translocation to dendrites is specific to certain mRNA species, or if there is a global increase in the trafficking of mRNAs upon neuron activity. Also, the exact molecular mechanism and potential factors involved in linking neuronal stimulation and mRNA localization are poorly understood.

Bidirectional movement of mRNAs in both anterograde and retrograde can occur in dendrites by simultaneous association of mRNAs with both Dynein and Kinesin. Bidirectional transport of *CamKIIα* granules can be explained by a study that showed cytoplasmic polyadenylation element binding protein (CPEB) interacts with *CamKIIα* 3′ UTR and can be co-immunoprecipitated with both Dynein and Kinesin-1 [20]. Overexpression of CPEB enhanced the localization of the mRNA to the dendrites [21], although it was not determined if this mechanism was through a direct effect on Dynein or Kinesin-1 activity.

Molecular motors play a significant role in active localization of mRNAs. The RNA binding protein (RBP) fragile X mental retardation protein (FMRP), which is present in ribonucleoprotein particle (RNP) granules [20], and is involved in regulating translation, can interact with both Kinesin heavy chain (KHC) and Dynein heavy chain (DHC) in *Drosophila* [22]. FMRP acts as a motor adaptor in mammalian cells, linking Kinesin light chain with mRNA to mediate stimulus-driven mRNA trafficking to dendritic spines [23],[24]. Overexpressing FMRP increased both the frequency and processivity of transporting mRNAs [25]. In contrast, downregulation of FMRP in *Drosophila* [26] and zebrafish [27] neurons increased arborisation, demonstrating that interfering with this RBP can result in morphological phenotypes. Stationary *ARC* mRNA molecules have been observed at the base of dendritic spines [28]. The base of dendritic spines can act as a hub for the accumulation of mRNAs, which can move to the spine upon request for local translation. The “sushi belt model” has been put forward to explain the relationship between bidirectional active movements of mRNAs and non-motile mRNAs at the synapses [29]. According to this model, a proportion of mRNAs is constantly moving into and out of the synapse and will be captured upon receiving a stimulus into a spine. Recent live imaging techniques that allow visualisation of translation of individual mRNA molecules in real time [30] can be used to study mRNA localization and translation dynamics in response to neuronal activation.

## 3. Functional importance of mRNA localization in different systems

Coupled with local translation, mRNA localization can provide several advantages compared to protein trafficking. Asymmetric distribution of mRNAs in the cytoplasm can provide neurons with both temporal and spatial resolution over the expression of proteins in different regions of cytoplasm, as translation of pre-localized messages can be triggered on demand. The ability to rapidly respond to different stimuli and regulate the expression level of the corresponding proteins can be crucial in highly polarised cells, such as neurons. Another potential advantage of targeting proteins by prelocalizing their RNAs is that proteins that are newly synthesized from localized mRNAs can have different functions compared to the existing repertoire of proteins in the cell, due to differences in their post-translational modifications (PTMs) [31]. For example, newly synthesised β-actin protein from localizing mRNAs is required for cell polarity and directional movement of fibroblasts [32]. However, the critical molecular distinction between old and new β-actin protein molecules is not known yet and requires further investigation.

In addition, direct transportation of some proteins, especially those that have a high binding affinity to cytoskeletal elements and other cytoplasmic factors, can pose logistically challenging for cells. In such cases, cells can take advantage of localizing translationally silenced mRNAs to their target sites, followed by triggering of translation at the destination. For example, Map2 and Tau proteins bind to microtubules and cannot readily be trafficked to the distal regions of the neurons; therefore, they are localized as precursor mRNAs to dendrites and axons, respectively before they are translated [33]. mRNA localization can be more energy-efficient than direct trafficking of individual proteins. This is because, in principle, multiple proteins can be synthesised from a single localized mRNA on-demand [3]. Finally, locating the elements that mediate mRNA localization in the untranslated regions (*UTR*) of mRNAs does not require any changes in the coding sequence of transcripts [2]. Thus, there are no additional constraints placed on the sequences used to code the protein.

## 4. mRNA localization in axons

Formation of a functional neuronal network requires precise positioning of axons in the CNS. Early studies failed to identify ribosomes in mature vertebrates' axons, leading to a notion that all axons do not have the capacity for local translation, and thus shifting the focus to dendritic mRNA trafficking [34]. However, landmark metabolic labelling studies showed that after isolating the soma, developing axons of invertebrates and vertebrates have the capacity to translate mRNAs [35]. Biochemical studies have shown that some of the components of the protein synthesis machinery, such as mRNAs, ribosomal RNAs and translating ribosomes, are present in the giant mature squid axons [36]. Various studies using electron microscopy *in vitro* and *in vivo* demonstrated that ribosomes were present in mammalian axons [37],[38]. However, it is still not known whether ribosomes are present in axons as monosomes or polysomes. Data from cultured neurons suggest that ribosomes might form polysomes in cultured cells [37], whereas monosomes might be the predominant species in neurons *in vivo* [39]. Using immuno-electron microscopy with genetically tagged ribosomes provided ultrastructural evidence for the presence of ribosomes in mature mammalian myelinated in axons was acquired [40].

A study by Tcherkezian et al. [41] could explain the lack of ribosome observation in axons to some extent by showing ribosomes were located very close to the plasma membrane, where they could associate directly with transmembrane (TM) receptors and were released into the axoplasm upon ligand binding and receptor activation. Tethering components of the translation machinery to the cytoplasmic domain of TM receptors allows spatial and temporal regulation of protein expression in response to external cues. Furthermore, electron microscopy of developing *Xenopus* retinal ganglion cells axons demonstrated that receptor-ribosome coupling is used by guidance cues where guidance cues can induce dissociation of ribosomes followed by specific mRNA translation [42].

For many years it was unclear whether vertebrate axons have the machinery to fold and process the locally synthesised proteins as various electron microscopy attempts failed to identify rough endoplasmic reticulum (RER) and the Golgi apparatus in axons [43]. The failure to identify the RER and the Golgi could be due to morphological properties of axons that are extended in shape. Thus, axons might have structures equivalent to the RER and the Golgi with similar functional properties. Consistent with this notion, fully folded proteins were synthesized and inserted into the plasma membrane in cultured isolated axons in the absence of soma [44]. Similarly, although isolated axons from molluscs (*Lymnea stagnalis*) do not have conventional RER and Golgi, injection of exogenous mRNAs into axons isolated from the soma resulted in the translation of fully functional transmembrane proteins [45]. Recent proteomic experiments demonstrated that about 350 proteins in isolated retinal axons are dynamically translated in response to different cues [46].

It is still unknown whether newly synthesized proteins in axons require specific post-translation modification compared to the proteins present in the soma, which might be achieved by the presence of RER and Golgi with a special function.

**Figure 3.**
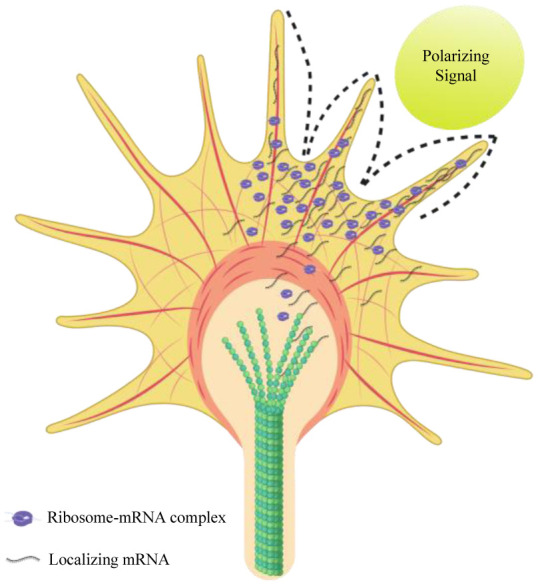
mRNA localization and translation in a developing growth cone in response to external cues. External cues can form a gradient of polarizing signal which induces localization of neuronal mRNAs important for cytoskeleton formation during axon guidance. The figure is based on the model proposed by Lin and Holt [51].

Extrinsic signals can affect growth, pathfinding, and synaptogenesis of developing axons (Figure 3). Mature axons are also dependent on extrinsic factors for maintenance and repair after damage [39]. *In vitro* data from cultured neurons have shown that isolated axons require axonal protein synthesis and degradation to respond to chemotropic factors or to regenerate after an induced injury (axon-severing) [47],[48]. Developing axons can localize mRNAs and synthesise proteins locally in their growth cones to assist in targeting the correct destination [49],[50].

Navigating axons can also grow in the absence of soma. The removal of the cell body did not affect *Xenopus* retinal axon pathfinding [52], indicating that translation machinery is available in the axons independent of the soma. Therefore, local protein synthesis in axons can potentially play an important role in both developing and mature axons. Mature axons also have the capacity for mRNA trafficking and protein synthesis for maintenance and regeneration after injury [49],[50].

**Figure 4.**
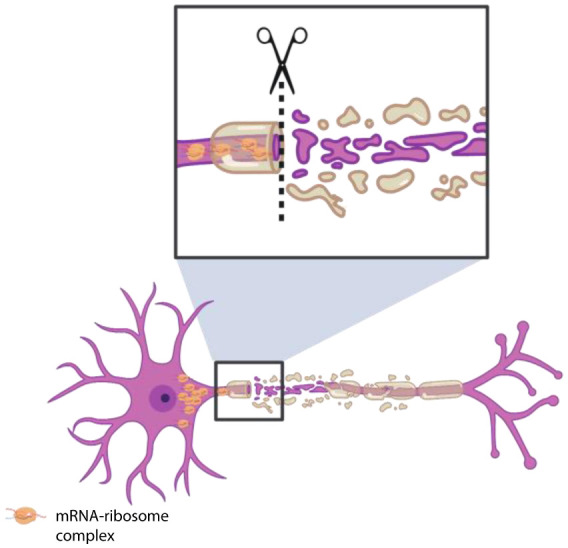
Elevated mRNA localization can occur in response to axonal injury. mRNA localization in response to injury can provide neurons with an enhanced capacity to repair and regenerate. Localization of mRNAs encoding important proteins in cell injury and repair is selectively increased following injury.

Microarray analysis combined with fluorescent *in situ* hybridization (FISH) showed that more than 300 mRNAs encoding various components of the translational machinery, cargo transportation, cytoskeleton and mitochondria are enriched in axons of the CNS and peripheral nervous system (PNS) of mammalian neurons [50]. In their study, healthy axons were capable of changing the composition of localizing mRNAs following axotomy by enriching mRNAs required for repair and regeneration [50]. While mRNAs encoding components of cargo trafficking, cytoskeleton and mitochondria showed a decrease in localization, there was an upregulation in localization of transcripts encoding proteins involved in axon targeting and synaptogenesis. These observations provided evidence for RNA localization-based adaptation of injured axons to regenerate and form synapses (Figure 4).

Axonal branching in the vertebrate CNS is linked closely to local protein synthesis. Guidance cues such as Netrin-1, BDNF, Sema3A, and Slit2 trigger local protein synthesis in the responsive neuron [48],[51],[53], and also function as modulators of arborisation [54]. For instance, the stimulus-dependent phosphorylation of the ZBP1 RBP by Src in response to BDNF resulted in ZBP1 dissociation from *β-actin* and translation induction, thereby promoting growth cone turning [55].

In cultured dorsal root ganglion (DRG) neurons, mRNAs encoding the components of the actin-nucleating Arp2/3 complex are localized to branch points and promote branch formation in response to nerve growth factor (NGF) [56]. Using high-resolution live imaging, it was shown that RNA granules localized to the sites of nascent branch formation in retinal ganglion cell (RGC) neurons in *Xenopus in vivo*, and move to the distal tip of stable branches [57]. Blocking *β-actin* translation disrupted branch formation, thereby demonstrating the importance of mRNA localization and local protein synthesis in arbour formation and stabilisation [57]. Thus, local synthesis of components of the actin cytoskeleton can provide the basis for branch formation on-demand. Interestingly, quantitative proteomic studies demonstrated that Arp2/3 complex, WAVE2, VASP and other actin binding molecules are enriched in cell protrusions, a role in agreement with their involvement in neuronal branch formation [58].

Deep sequencing of ribosome-bound mRNAs showed that many presynaptic proteins are translated locally in mouse retinal axons during branch formation [59]. The degree of presynaptic arborisation can determine the number of post-synaptic connections that a neuron can have, hence defining the complexity of neural circuits formation in the CNS [60],[61]. Hence, local protein synthesis in axons can have a fundamental role in synaptogenesis and neural circuit assembly.

Defective assembly of RNPs has been implicated in the pathogenesis of neurological diseases that affect axons such as amyotrophic lateral sclerosis (ALS) and spinal muscular atrophy (SMA) [62]. Survival motor neuron (SMN) protein regulates localization and local translation of *β-actin*, *growth-associated protein 43* (*GAP43*) and *neuritin/cpg15* mRNAs in motor neurons [62],[63]–[68]. Overexpression of SMN-interacting proteins HuD and ZBP1 rescued the reduced translation of the interacting mRNAs in the growth cone [68]. Thus, there is compelling evidence that mRNA trafficking in axons plays an important role in axonal development, maintenance and disease. However, the mechanisms involved in linking axonal mRNAs to molecular motors and the RNA localization signals required for axonal mRNA targeting are still poorly understood. A mechanistic understanding of RNA localization in axons may provide us with information on mechanisms of neurological diseases.

## 5. Conclusion

Localization of mRNA and subsequent translation can provide intricate network of the neurons with a delicate control over gene expression in different compartments at different times. Various studies have demonstrated the functional importance of mRNA localization in axon and dendrites. While molecular mechanism of mRNA localization is conserved in neurons from invertebrates to vertebrates, the role of different factors and regulators in trafficking and translation of mRNAs remains to be discovered. Although *in vitro* studies of cultured neurons have provided very useful information on mRNA localisation and local translation, we still do not have a clear understanding of the detailed molecular mechanisms underlying axonal mRNA localisation. This is particularly the case *in vivo*, where neurons are present in their physiological environment. Further studies are required to decipher the role of neuronal activity in mRNA localization during formation of neuronal network.
